# DELETION OF EPE1 GENE ENHANCES LONGEVITY IN SCHIZOSACCHAROMYCES POMBE

**DOI:** 10.51894/001c.123097

**Published:** 2024-08-30

**Authors:** Yongqi Xu

**Affiliations:** 1 College of Osteopathic Medicine Michigan State University

## 83

Aging is a biological process that occurs in all living organisms and is marked by gradual reductions in physiological functions. On a cellular level, this complex process is known to be associated with epigenetic changes. But it remains unclear how epigenetic changes impact the aging process. Using the fission yeast, Schizosaccharomyces pombe, as an aging model, we discovered that chromosomal deletion of a gene called epe1 (epe1∆) extends aging. This is interesting because the human homolog of yeast epe1 (called KDM2B) is also associated with aging and cellular senescence. Epe1 is known to be important for stabilizing and preventing aberrant spreading of an epigenetic state called heterochromatin, which represses underlying gene expression. We hypothesized that the slowed aging phenotype of epe1∆ mutant cells is related to heterochromatin regulation. We tested this by generating double genetic mutants that can restore either of the known heterochromatin functions of Epe1. Specifically, in epe1∆ cells, we introduced the additional deletion of either elp1 or clr4 gene to rescue heterochromatin stability or to abolish improper heterochromatin spreading, respectively. Surprisingly, our aging experiments revealed a lack of normalized aging in both of our double mutant cells. Future work will investigate whether Epe1 might have additional functions, outside of heterochromatin regulation, that is significant for the aging process. We envision that uncovering how the conserved epe1 gene regulates aging in S. pombe will inform us of how KDM2B may control similar processes in human. This will ultimately improve our understanding of human health and aging.

